# Regulation of Cellular Metabolism through Phase Separation of Enzymes

**DOI:** 10.3390/biom8040160

**Published:** 2018-12-03

**Authors:** Manoël Prouteau, Robbie Loewith

**Affiliations:** 1 Department of Molecular Biology, University of Geneva, 30 Quai Ernest-Ansermet, CH1211 Geneva, Switzerland; manoel.prouteau@unige.ch; 2 Institute of Genetics and Genomics of Geneva (iGE3), University of Geneva, 30 Quai Ernest-Ansermet, CH1211 Geneva, Switzerland; 3 Swiss National Centre for Competence in Research (NCCR) in Chemical Biology, University of Geneva, Sciences II, Room 3-308, 30 Quai Ernest-Ansermet, CH1211 Geneva, Switzerland

**Keywords:** phase separation, molecular condensates, protein filaments, metabolism, signalling

## Abstract

Metabolism is the sum of the life-giving chemical processes that occur within a cell. Proper regulation of these processes is essential for all organisms to thrive and prosper. When external factors are too extreme, or if internal regulation is corrupted through genetic or epigenetic changes, metabolic homeostasis is no longer achievable and diseases such as metabolic syndrome or cancer, aging, and, ultimately, death ensue. Metabolic reactions are catalyzed by proteins, and the in vitro kinetic properties of these enzymes have been studied by biochemists for many decades. These efforts led to the appreciation that enzyme activities can be acutely regulated and that this regulation is critical to metabolic homeostasis. Regulation can be mediated through allosteric interactions with metabolites themselves or via post-translational modifications triggered by intracellular signal transduction pathways. More recently, enzyme regulation has attracted the attention of cell biologists who noticed that change in growth conditions often triggers the condensation of diffusely localized enzymes into one or more discrete foci, easily visible by light microscopy. This reorganization from a soluble to a condensed state is best described as a phase separation. As summarized in this review, stimulus-induced phase separation has now been observed for dozens of enzymes suggesting that this could represent a widespread mode of activity regulation, rather than, or in addition to, a storage form of temporarily superfluous enzymes. Building on our recent structure determination of TOROIDs (TORc1 Organized in Inhibited Domain), the condensate formed by the protein kinase Target Of Rapamycin Complex 1 (TORC1), we will highlight that the molecular organization of enzyme condensates can vary dramatically and that future work aimed at the structural characterization of enzyme condensates will be critical to understand how phase separation regulates enzyme activity and consequently metabolic homeostasis. This information may ultimately facilitate the design of strategies to target the assembly or disassembly of specific enzymes condensates as a therapeutic approach to restore metabolic homeostasis in certain diseases.

## 1. Regulation of Metabolite Flux

Homeostatic control over metabolism requires intricate regulation of metabolite flux. Such regulation is important; Otto Warburg noted already in 1924 [1,2] that alterations in metabolic control are associated with cell transformation. Over the years we have learned that regulation of metabolic enzymes is achieved through many different mechanisms. These are nicely illustrated by the multiple modes of regulation of pyruvate kinase [3]. Pyruvate kinase catalyzes the conversion of phosphoenolpyruvate (PEP) and adenosine diphosphate (ADP) to pyruvate and adenosine triphosphate (ATP) in the final step of glycolysis. One mechanism of regulation of this conversion is the choice of pyruvate kinase isoform to be expressed. In mammals, there are four isoforms, PKM1 and PKM2, encoded by the *PKM* locus, and PKR and PKL, encoded by the *PKLR* locus. Expression of these isoforms is regulated both transcriptionally and post-transcriptionally through alternative splicing and correlates with metabolic demand of the tissue; PKM2 is expressed in proliferating and tumor cells while PKM1 is found in tissues with high catabolic demand such as heart and brain as well as some tumors [4]. In yeast there are two pyruvate kinase paralogs, *CDC19* and *PYK2*, whose expression is determined by glucose availability and thus associated with either fermentation (Cdc19) or respiration (Pyk2) [5]. 

Allosteric interactions permit a second, more acute regulation of pyruvate kinase activity to contemporaneously couple enzyme activity with cellular demand [3]. The best understood allosteric regulator of pyruvate kinase is the glycolytic metabolite fructose-1,6-bisphosphate (FBP). It activates both PKM2 and Cdc19 by stabilizing their conversion from modestly active dimers to robustly active tetramers. As evidenced in bacteria [6], this regulation plays an important role in controlling glycolytic flux; FBP accumulates when glucose is plentiful and signals a sufficiency, or full reservoir, of upper-glycolysis metabolites that, by stimulation of pyruvate kinase, can be siphoned off through lower glycolysis. In addition to FBP, pyruvate kinase is also regulated by other metabolites, including several amino acids [3]. This regulation presumably coordinates the siphoning off of upstream glycolytic intermediates which are necessary feedstock for other biosynthetic pathways. 

A third means by which pyruvate kinase activity is regulated is by post-translational modifications mediated by intracellular signaling pathways [3]. Cdc19 is phosphorylated on multiple residues in yeast cells [7,8,9]. Some of these phosphorylation events are mediated by Protein Kinase A (PKA) suggesting that Cdc19 activity could be mediated downstream of glucose-regulated cyclic adenosine monophosphate (cAMP) signaling although evidence for this is lacking [10]. Intriguingly, phosphorylation of several residues in a low-complexity region (LCR), by an unknown kinase, was recently suggested to alter Cdc19 activity through a fourth mode of regulation—phase separation into a molecular condensate [11].

Phase separation of Cdc19 is triggered by stresses including glucose starvation and heat shock and is readily apparent by the formation of bright foci in cells expressing GFP-tagged Cdc19 protein. In good growth conditions, the LCR of Cdc19 is either sequestered within the core of active tetramers or phosphorylated when solvent-exposed in monomers and dimers. Under stress conditions, the LCR is exposed and this is necessary and sufficient to drive condensation (phase separation) of Cdc19 into foci. Foci formation protects Cdc19 from stress-induced degradation. Upon return to favourable growth conditions, the LCR is phosphorylated which facilitates dissolution of the foci which is necessary for the restart of cell growth.

Phase separation of proteins has been known for a long time. Although not usually thought of in the context of phase separation, textbook examples include the transition of globular actin and α and β tubulin dimers into filamentous actin and microtubules respectively. The mechanical roles that phase separation of these and other structural proteins play in cell architecture and migration are obvious. Recently, it has become clear that many metabolic enzymes and other proteins with no obvious cytoskeletal roles also experience phase separations. In this review we will highlight some of the now many examples of metabolic enzymes that undergo phase separation in response to nutrition and other stresses. As noted by others [12,13,14,15], their sheer number suggests that phase separation represents a frequently used mechanism to control biochemical activity of these enzymes and thus metabolic homeostasis. We will also describe the various molecular architectures that these molecular condensates may acquire. In some circumstances, the formation of these condensates is reversible, as in the case of Cdc19 described above, while in others they are not. It is generally believed that reversible condensate formation allows cells to maintain a reserve of inactive enzymes that can be quickly remobilized upon return to favorable growth conditions. Our own recent work, as well as work from others, suggests that some of these condensates are well-organized polymers that mediate acute changes in protomer activity [16]. Elucidating the structures of these particular condensates provides detailed molecular insight into how phase separation can regulate enzymatic activity and may also guide future efforts into the design of approaches to manipulate this mode of regulation for therapeutic gain.

## 2. Metabolism-Related Enzyme Condensates in Yeast and Other Organisms

Using libraries of fluorescently tagged proteins, particularly in budding yeast, several groups have identified dozens of proteins that coalesce into foci upon acute starvation or in cells grown into stationary phase [17,18,19]. These foci range in their size and shape; some are spherical with diameters on the order of 10’s of nanometers whereas others are elliptical or filamentous with dimensions on the order of microns. In some cases, the proteins that form these condensates can be separated by ultra-centrifugation suggesting that these foci are rather solid objects within the cytoplasm. As summarized in Table 1, and discussed briefly below, many of these proteins are well-conserved metabolic enzymes. 

## 3. Phase Separation as a Means to Acutely Regulate Enzymatic Activity

The molecular triggers of these stimulus-dependent phase separations are generally poorly understood, and, in many cases, the structural organization of the components making up these foci is unknown. The lack of such structural information makes it difficult to appreciate how foci formation is induced, for example, by phosphorylation or changes in pH, and, additionally, to ascertain the functional consequences that foci formation has on the activities of the contained enzymes (Table 1). Indeed, often, for lack of a better explanation, foci are simply concluded to serve as macromolecular storage depots. However, the structures of a growing number of foci have now been examined. The current take-home message from these studies is that the molecular architectures of cellular foci vary along a continuum, from weakly interacting particles that form fluid condensates (liquid droplets) to strongly interacting particles that form rigid condensates (partially organized amyloids or highly organized polymers). To date, most attention in the phase separation field has focused on liquid droplets and amyloids. As these types of condensates have been reviewed extensively [42,43,44] we will mention them only in passing here, focusing instead on polymeric condensates.

**Figure 1:**
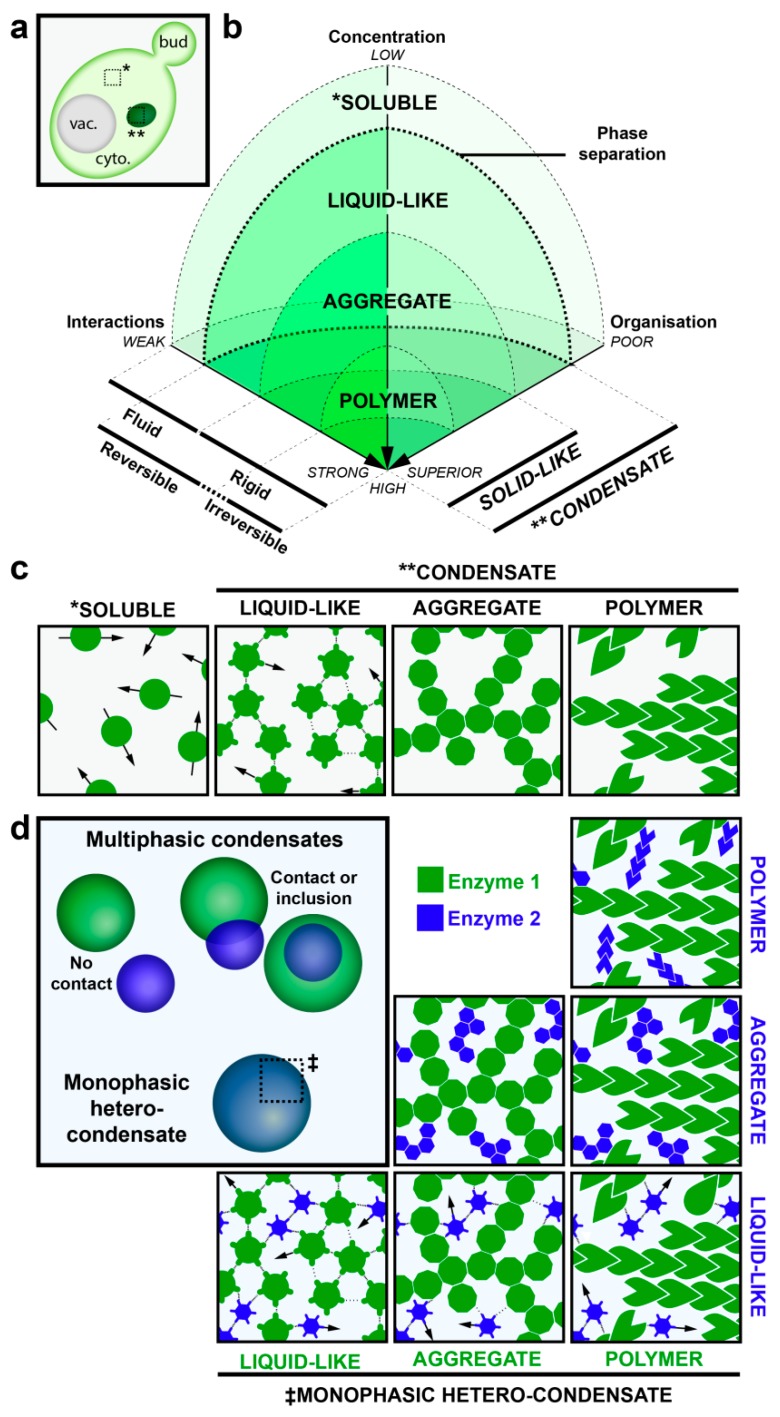
Biophysical properties of protein condensates. (**a**) Schematic representation of phase separation of proteins in yeast. Phase separation allows components to undergo a transition from soluble (*), diffuse localization to one or a few molecular condensates (**) that appear as foci in light microscopy [43,44]. (**b**,**c**) This phase transition and the type of condensates depend on three component properties: The concentration, the strength of component-component interaction and the intrinsic organization. Phase transition occurs when components reach a critical saturation due to increases of local concentrations and/or augmentation of component-component interactions relative to solvent-component interactions. Weak electrostatic intermolecular interactions are more likely to lead to liquid-like droplets which require little energy input to dissolve and thus components in these condensates are quite mobile. Stronger intermolecular interactions generate solid-like foci which require a high energy input to dissolve and thus components of these condensates acquire a more rigid aspect. The level of organization within the solid-like condensates distinguishes aggregates, unorganized condensates, from polymers, which are well organized. (**d**) Condensates can contain multiple components (hetero-condensate) and can possess different molecular organizations (multi-phasic, ‡), as illustrated.

- Liquid Droplets: The phase separation continuum is illustrated in Figure 1. Condensates referred to as liquid droplets are composed of one or more factors that self-associate through numerous, weak interactions. Consequently, the factors within these condensates are highly mobile and can be rapidly exchanged with the soluble phase as assessed by fluorescence recovery after photobleaching. RNA- and protein-containing P-granules are the prototypic example of this type of condensate. The proteins that form liquid droplets typically contain domains of low sequence complexity known as inherently disordered regions.
- Amyloid-like aggregates: Further concentration of these proteins, combined with partial unfolding of the inherently disordered regions, can lead to rigidification of the condensate into what can be referred to as an aggregate. Relative to liquid droplets, proteins in aggregates are held together by stronger β-strand-like contacts that form amyloid fiber-like structures. In amyloid-fiber-like condensates/aggregates, proteins are much less mobile and typically present only a partially organized structure (Figure 1). Cdc19 condensates, described above, have been proposed to be reversible amyloid-like aggregates [11], while other amyloid-fiber-like aggregates appear to be more stable and even engaged in irreversible, potentially pathogenic aggregates that can only be cleared through degradative pathways [45,46,47].
- Polymers: Electron microscopy (EM) studies initiated in the 1970s revealed that many metabolic enzymes form filaments and/or helices [20,29,30,36,37,39,40,41,48]. These are typically formed upon exposure to an extrinsic stimulus, suggesting that enzyme polymerization represents an underappreciated mechanism by which cells regulate enzymatic activity and metabolic homeostasis. Polymers thus potentially represent another type of rigid protein condensate found in cells. Unlike aggregates, polymers would present a well-defined structure that can be readily disassembled in vivo. Indeed, rapid assembly/disassembly kinetics would endow upon this type of condensate the potential to acutely regulate enzymatic activity (Figure 1). We highlight now a selection of metabolic enzymes that appear to be regulated through phase separation into polymers.

### 3.1. Examples of Phase Separation in Carbohydrate Metabolism

Glycolysis plays a central role in metabolism, providing energy as well as carbon feedstocks for anabolic pathways. As illustrated in Table 1, several glycolytic enzymes were found to condense into foci upon exposure to various stresses [17,19].

The condensation of Cdc19 upon starvation was described above. Interestingly, starvation-induced Cdc19 foci also contain the stress granule marker Poly(A) binding protein Pab1 and mRNA. Stress granules contain mRNAs and translation initiation factors and are hypothesized to serve as a cache of cellular components that will later be used to kick-start regrowth following stress relief [11]. However, Cdc19 in yeast, as well as in mammalian hepatocytes, also condenses into foci upon exposure to hypoxia [22]. These particular Cdc19 foci, which form under very different metabolic conditions in the cell, were referred to as G bodies and were found to additionally contain phosphofructokinases Pfk1 and Pfk2, enolase Eno2 and Fructose 1,6-bisphosphate aldolase Fba1 as well as chaperones but no components typically associated with stress granules. Collectively, these studies demonstrate that different stresses can trigger the formation of monophasic hetero-condensates (Figure 1d) composed of multiple enzymes (and mRNA) depending on the stress that led to their formation. 

**Figure 2:**
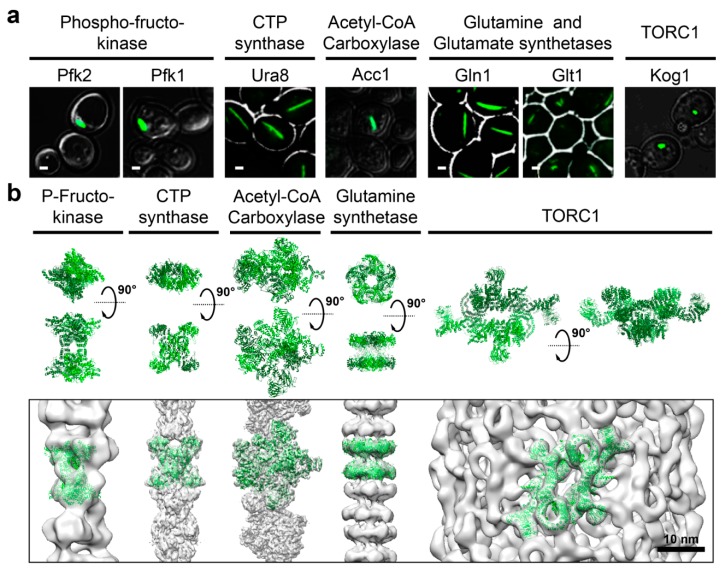
Metabolism related enzymes form polymers in various organisms. (**a**) Examples of metabolic enzymes observed to coalesce into cytosolic condensates. Adapted from Shen et al. (2016) [19] and Petrovska et al. (2014) [38]. (**b**) Structures of metabolic enzymes that polymerize into filaments. The protomer of the polymer is shown above, and placed into the filament below. From the Protein Data Bank: Model of P-Fructo-Kinase PDB ID 4XYJ [61], model of cytidine triphosphate (CTP) synthase PDB ID 5U03 [51], model of Acetyl-CoA 6G2D [33], model of Glutamine synthetase PDB ID 3FKY [53], model of mTORC1 PBD ID 5FLC [62]. From the Electron Microscopy Data Bank: Negative stain electron microscopy (EM) map of P-Fructo-Kinase filament emd-8542 [21], cryo-EM map of the human CTP synthase filament emd-8474 [51], cryo-EM map of human Acetyl-CoA with citrate emd-4342 [33], model of cryo-EM map filtered at 20 Ang of the yeast Glutamine synthetase filament based on the dodecameric oligomer [53], cryo-EM map of TOROID emd-3814 [16].

Purification of the glycolytic enzyme ATP-dependent 6-phospho-fructokinase (PFK) from pig liver demonstrated that it assembles into high molecular weight, potentially polymeric, structures [49]. Subsequent EM studies confirmed that PFK functions as a tetramer which can assemble into filaments in vitro [20], particularly in the presence of its substrate fructose 6-phosphate [21] (Figure 2). Florescence microscopy of GFP-tagged PFKL shows that the enzyme condensates are present in vivo, and hyper accumulate upon addition of citrate, an allosteric inhibitor of PFK1. These observations suggest that PFKL puncta correspond to a polymerized form of the enzyme and that polymerization serves to acutely regulate activity [21]. The observation that yeast Pfk1 also coalesces with other glycolytic enzymes into G bodies [22] could suggest that these monophasic hetero-condensates have a complex, potentially multiphasic, organization. Determining the structure of G bodies will be highly informative, shedding light on if and how polymerization of Pfk1 with the condensation other enzymes is regulated to control glycolytic flux. This will also help to understand how Cdc19 can condense into structurally different types of cytosolic foci depending on the upstream cue.

### 3.2. Examples of Phase Separation in Nucleotide Metabolism

Nucleotide metabolism-related enzymes can be fundamentally classified into pyrimidine and purine biosynthesis pathways (Table 1). In both prokaryote and eukaryote cells, several of the enzymes catalyzing these reactions form cytoplasmic condensates in response to specific nutrient cues. 

Cytidine triphosphate (CTP) synthase is a key enzyme in pyrimidine biosynthesis catalyzing the ATP-hydrolysis-dependent conversion of uridine triphosphate and glutamine to CTP and glutamate. Normally a tetramer, this enzyme has been observed to form cytoplasmic structures of varying shapes in *Escherichia coli* [23,50,51], yeast [3,18], the drosophila egg chamber and female germline [24,25,52] and human cells [50,51]. Cryo-electron microscopy examination of bacterial and human CTP synthase demonstrated that these enzymes polymerize into filaments [26,51]. Polymerization of the human enzyme is promoted by addition of substrates (Uridine triphosphate and ATP) and disfavored by addition of products (CTP and ADP). Consistently, polymerization of the human enzyme was found to increase enzymatic activity. Assuming that the CTP synthase condensates observed in vivo (Figure 2) correspond to the polymerized, and thus active, form of the enzyme, argues against the widely held notion that foci universally represent storage depots of inactive enzymes.

Similarly, enzymes required for de novo purine biosynthesis form cytoplasmic foci in various eukaryote cells under starvation conditions [17,25,28] (Table 1). De novo purine biosynthesis begins with phosphoribosyl pyrophosphate, requires 10 enzymatic conversions catalyzed by six different enzymes, and culminates with the production of inosine monophosphate, the precursor of guanosine and adenosine monophosphate. These six enzymes undergo profound intracellular spatial reorganization upon stresses such as purine deprivation or starvation. In yeast, PPAT (Ade4) [17], GART (Ade5/7) [17,25,28], FGAMS (Ade6) and ATIC (Ade17) orthologues form puncta in quiescent cells [17,28] (acronyms are expanded in the legend of Table 1). In addition, GART and PAICS drosophila orthologues assemble into large-scale intracellular structures in the female germline [25]. In HeLa cells, acute purine-deprivation induces a quick but reversible localization of all de novo purine biosynthesis enzymes into a monophasic hetero-condensate called purinosomes [27,28]. The molecular organization of the purinosome is unknown and its function remains speculative—it is tempting to think that its formation would permit more efficient molecular flux in this multistep biosynthetic pathway. 

### 3.3. Examples of Phase Separation in Fatty Acid Metabolism

Fatty acids serve as cellular energy reserves, the building blocks of membrane lipids and as signaling molecules. Fatty acid biosynthesis begins with the ATP-dependent carboxylation of acetyl-CoA by acetyl-CoA carboxylase to yield malonyl-CoA unto which additional acetyl groups are added by fatty acid synthase (FAS) until the final product, palmitate, is finally produced. Fatty acid biosynthesis needs to be tightly regulated—aberrant accumulation of fatty acids is associated with many common diseases including diabetes, insulin-resistance, and cancer. 

Acetyl-CoA carboxylase (ACC), fatty acid synthase and many other enzymes involved in lipid homeostasis have been shown to form condensates [31]. In yeast, GFP-tagged Acetyl-CoA carboxylase (ACC1), particularly upon starvation, condenses into large, elongated structures [19,31] (Figure 2). In animal cells, ACC condensates are induced upon exposure to citrate [29,32,34,35]. It has been known for 50 years that ACC forms filaments in vitro and in vivo [29,30,32,33,34,35,48]. Recently, structures of human ACC1 filaments were described [33]. In the presences of citrate, ACC1-citrate dimers assemble into a polymer with high enzymatic activity. In contrast, in the presence of palmitoyl-CoA dimers within the polymer become locked in an inactive conformation. This work again challenges the assumption that filaments/condensates are storage forms of inactive enzymes; and, furthermore, demonstrates that enzymes can polymerize into different higher-order structures, with different functional outcomes, according to different upstream stimuli.

Additional enzymes involved in lipid metabolism have been observed to form condensates. Fas1 and Fas2 subunits of fatty acid synthase (FAS) have been shown to co-sequester into foci in stationary-phase cells [17,31]. Phospholipid (Phosphatidylinositol synthase, Pis1) and ergosterol biosynthesis (Delta-sterol C-methyltransferase, Erg6) also condense into different cellular compartments, distinct from the FAS complex [31]. These condensates are presently thought to serve as storage depots of inactive enzymes; their intrinsic organization remains to be investigated.

### 3.4. Examples of Phase Separation in Amino Acid Metabolism 

Amino acids play many roles in cellular and organismal metabolism. They are the building blocks of proteins but serve additional, nonproteinogenic roles as well, for example, as precursors for the biosynthesis of purines and pyrimidines, the neurotransmitter gamma-amino-butyric acid (from glutamate), the vasodilator nitric oxide (from arginine), iron-binding porphyrins (from glycine), etc. Fluorescent microscopy screens of yeast cells expressing GFP-tagged proteins revealed cytosolic assemblies of several enzymes involved amino acid metabolism (Table 1, Figure 2). These include, asparagine synthase [19], glutamate synthetase [18], glutamate dehydrogenase [19], glutaminase [41] and glutamine synthetase [17,38]. Once again, these puncta are generally assumed to represent storage depots for these enzymes. 

Among these enzymes, the most studied is glutamine synthetase which exists as a decameric complex that can polymerize into elongated filaments upon starvation-induced decrease in the intracellular pH [38,40]. In yeast, filamentation of glutamine synthetase leads to its inactivation. Mutations that prevent filamentation prevent quiescent cells from properly restarting growth but the mechanistic details behind this phenotype are unclear as only a low-resolution structure of glutamine synthetase filaments has been reported [40]. The enzyme is known to form dodecamers in vitro [53] which seem to be the basis (protomers) of longer polymers in vivo [38] (Figure 2). Other amino acid biosynthetic enzymes known to form filaments in vitro include cystathionine β-synthase [54] and glutaminase [41]. Cystathionine β-synthase converts serine into cysthationine (the precursor of the cysteine). This enzyme functions as a homo-tetramer which phase coalesces into foci in starved yeast [17] which presumably correspond to a polymeric form of the enzyme that has been observed in vitro [54]. The effect of polymerization on activity is unknown. Glutaminase in pig renal cells also forms polymers in vitro [41]. These polymers contain active enzymes but their structure is also unknown.

In summary, there are mounting numbers of metabolic enzymes that form molecular condensates in vivo, particularly under starvation or other stressful conditions. A large subset of these enzymes is additionally known to form polymers in vitro. Although validated in very few cases, it seems likely that in many, if not most cases, the condensates formed in vivo correspond to polymerized forms of the enzymes. Structure determination of these polymers is important is this will reveal how these enzymes are regulated.

## 4. Metabolism-Related Signaling Enzymes Also Polymerize/Coalesce into Foci

In addition to the metabolic enzymes noted above, signaling enzymes that regulate metabolic homeostasis are now also known to reorganize into molecular condensates upon exposure of cells to various stresses. The ser/thr protein kinase Target of Rapamycin Complex 1 (TORC1) is one such enzyme. 

TORC1 is a mega-Dalton-sized complex of four proteins, each present in two copies. In the presence of adequate nutrients, and in the absence of noxious stressors, TORC1 is active generating signals that both support anabolic processes (e.g., protein, lipid and nucleotide biosynthesis) and suppress catabolic processes such as autophagy [55]. Under these pro-growth conditions, TORC1 is diffusely localized to the vacuolar membrane in yeast or the lysosomal membrane in mammalian cells. However, upon application of various abiotic stresses or upon limitation of certain nutrients, both yeast and mammalian TORC1 have been observed to relocalize into discrete condensates [17,56,57,58,59,60]. The best characterized of these structures is the punctum formed by yeast TORC1 upon depletion of glucose from the medium [16]. Analysis of these condensates using super-resolution optical microscopy and 3D reconstructions of cryo-electron micrograph revealed that TORC1 organizes into a helical structure called a TOROID (TORC1 Organized in Inhibited Domain; Figure 2) [16]. TORC1 assembly into a TOROID physically occludes the kinase pocket and thus serves to arrest TORC1 signaling by hindering substrate access to active site. Readdition of glucose triggers rapid disassembly of the TOROID concomitant with reactivation of TORC1 kinase activity. This regulation appears to require the RAG family GTPases Gtr1 and Gtr2. Presumably the nucleotide binding status of these GTPases is regulated by an unknown glucose metabolite. How changes in nucleotide loading translates into TORC1 polymerization/depolymerization remains completely unknown and a fascinating question for the future. Thus, TOROID formation serves as an elegant way to acutely inactivate this kinase and to cache it for later fast reactivation upon return of favorable growth conditions. Thus, not only metabolic enzymes, but also enzymes that signal to the metabolic machinery are regulated through reversible polymerization.

## 5. Outlook

As elaborated in this review, it is now apparent that many metabolic enzymes undergo a phase separation into molecular condensates in response to various stimuli. We argue that, in many cases, the condensates formed represent highly-organized enzyme polymers. In some cases, the structure and activity of the polymerized enzymes have been investigated, revealing that polymerization can lead to either activation or inhibition of activity. Thus, molecular condensates cannot automatically be assumed to represent storage depots of surplus enzyme. 

Determination of the molecular organization of enzyme condensates will be highly rewarding. Ideally, these determinations should be done both in vitro and in vivo. Recent advances in cryo-EM and cryo-electron tomography make these lines of investigation quite feasible. High resolution structure determinations will help us understand the molecular triggers that lead to enzyme condensation and will reveal the functional consequence that condensation has on enzyme activity. This knowledge will potentially yield insight into how this mode of enzyme regulation is corrupted in disease and how it can be targeted for therapeutic gain.

We note that many of the enzymes that form higher-order polymers are found in stabile oligomeric (e.g., ACC1) or hetero-oligomeric (e.g., TORC1) structures when not polymerized. Interestingly, simple overexpression of oligomeric enzymes (or slightly modified variants thereof) often leads to their polymerization [63]. This suggests that many oligomeric enzymes have a natural propensity to polymerize and that the balance between protomer and polymer is easily tipped (for example by change in pH, through a post-translational modification, or through binding of an allosteric regulator). Thus, in addition to catalytic activity, the ability to form oligomers was likely also an attribute under evolutionary selection.

Lastly, we anticipate that additional study will reveal intriguing properties of enzyme condensates. Of particular interest are the enzymes that adopt different condensate architectures depending on the upstream stimulus—active (citrate-bound) and inactive (palmitate-bound) polymers of ACC1 provide evidence for such regulation. More complex variations on this theme would be the formation of different hetero-condensates involving the co-assembly of different enzymes. Again, evidence for such structures exist including G-bodies and purinosomes [64]. 

Only with an appreciation of these structures will we understand how cells achieve metabolic homeostasis.

## Figures and Tables

**Table 1:** Summary of metabolism-related enzymes observed to form condensates in bacteria (B), yeast (Y), drosophila (D) and mammals (M). Note that many of these enzymes function as oligomers, an attribute that likely contributes to their ability to form higher-order structures. ADSL: Adenylosuccinate lyase; ATIC: 5-aminoimidazole-4-carboxamide ribonucleotide transformylase and inosine monophosphate cyclohydrolase; CAD: carbamoyl-phosphate synthetase 2, aspartate transcarbamylase and dihydroorotase; FGAMS: Formylglycinamidine-ribonucleotide synthetase; GART: glycinamide ribotide and aminoimidazole ribotide synthetases; PAICS: Phosphoribosylaminoimidazole carboxylase; PPAT: Phosphoribosylpyrophosphate amidotransferase. Stresses that trigger condensate formation in yeast: Stationary phase (SP), glucose starvation (GS), purine starvation (PS), acidic pH (apH), heat shock (HS) and hypoxia (HY).

Metabolic Functions		Enzyme Name	Protomer	Condensates				Yeast		References
				*B*	*Y*	*D*	*M*	Gene Name	Stress Triggering Condensation	
**Carbohydrate metabolism**		Glycogen debranching enzyme			☑			**Gdb1**	SP	[19]
		ATP-dependent 6-phosphofructokinase	Tetramer		☑		☑	**Pfk1/2**	SP	[19,20,21]
		Pyruvate kinase	Tetramer		☑		☑	**Cdc19**	SP, GS, HS	[11,17]
		Enolase	Dimer		☑			**Eno2**	HY	[22]
		Fructose bisphosphate aldolase			☑			**Fba1**	HY	[22]
		Alcool deshydrogenase	Tetramer		☑			**Adh2**	SP	[17]
		UTP-glucose-1-phosphate uridylyltransferase	Dimer to octamer		☑			**Ugp1**	SP	[17]
**Nucleotide metabolism**	**Pyrimidine synthesis**	CTP synthase	Dimer and tetramer	☑	☑	☑	☑	**Ura7/8**	SP, GS	[18,23,24,25,26]
		Trifunctional CAD enzyme	-		☑			**Ura2**	SP	[17]
		Adenylosuccinate synthetase	Dimer		☑			**Ade12**	SP	[17]
	***De novo* purine synthesis**	PPAT enzyme	Tetramer		☑		☑	**Ade4**	SP	[17,27]
		Trifunctional GART enzyme	Dimer		☑	☑	☑	**Ade5/7**	SP, PS	[17,25,27,28]
		Bifunctional PAICS enzyme	Octamer		☑	☑	☑	**Ade2**	-	[25,27]
		FGAMS enzyme	Monomer		☑		☑	**Ade6**	-	[17,27,28]
		ADSL enzyme	Tetramer				☑	**Ade13**	-	[27,28]
		Bifunctional ATIC enzyme	Dimer		☑		☑	**Ade17**	SP, PS	[17,27,28]
**Fatty acids and Sterol metabolism**		Acetyl-CoA carboxylase	Dimer and tetramer		☑		☑	**Acc1/2**	SP, GS	[19,29,30,31,32,33,34,35]
		Fatty acid synthase complex	-		☑			**Fas1/2**	SP, GS	[17,31]
		Sterol 3-beta-glucosyltransferase	-		☑			**Ugt51**	SP	[17]
**Amino acids metabolism**		Asparagine synthetase	Dimer		☑			**Asn1/2**	SP	[19]
		Glutamate synthetase	Hexamer		☑			**Glt1**	SP	[18,19,36,37,38]
		Glutamate dehydrogenase	Hexamer		☑		☑	**Gdh2**	SP	[19,39]
		Glutamine synthetase	Decamer	☑	☑			**Gln1**	GS + apH	[17,19,38,40]
		Glutaminase	Tetramer			☑		**-**	-	[41]
		Cystathionine beta-synthase	Tetramer		☑			**Cys4**	SP	[17]
**Metabolism regulator**		Target of Rapamycin Complex 1	Dimer of heterotetramer		☑			**TORC1**	SP, GS	[16]
